# Stress sharing as cognitive glue for collective intelligences: A computational model of stress as a coordinator for morphogenesis

**DOI:** 10.1016/j.bbrc.2024.150396

**Published:** 2024-10-30

**Authors:** Lakshwin Shreesha, Michael Levin

**Affiliations:** a Department of Biology, Tufts University, Medford, MA, 02155, USA; b Allen Discovery Center at Tufts University, Medford, MA, 02155, USA; c Wyss Institute for Biologically Inspired Engineering, Harvard University, Boston, MA, 02115, USA

**Keywords:** Swarm intelligence, Cells, Embryos, Development, Morphogenesis, Stress

## Abstract

Individual cells have numerous competencies in physiological and metabolic spaces. However, multicellular collectives can reliably navigate anatomical morphospace towards much larger, reliable endpoints. Understanding the robustness and control properties of this process is critical for evolutionary developmental biology, bioengineering, and regenerative medicine. One mechanism that has been proposed for enabling individual cells to coordinate toward specific morphological outcomes is the sharing of stress (where stress is a physiological parameter that reflects the current amount of error in the context of a homeostatic loop). Here, we construct and analyze a multiscale agent-based model of morphogenesis in which we quantitatively examine the impact of stress sharing on the ability to reach target morphology. We found that stress sharing improves the morphogenetic efficiency of multicellular collectives; populations with stress sharing reached anatomical targets faster. Moreover, stress sharing influenced the future fate of distant cells in the multi-cellular collective, enhancing cells’ movement and their radius of influence, consistent with the hypothesis that stress sharing works to increase cohesiveness of collectives. During development, anatomical goal states could not be inferred from observation of stress states, revealing the limitations of knowledge of goals by an extern observer outside the system itself. Taken together, our analyses support an important role for stress sharing in natural and engineered systems that seek robust large-scale behaviors to emerge from the activity of their competent components.

## 1 Introduction

Morphogenesis, broadly considered, is the ability of groups of cells to build complex, functional anatomical structures. Large-scale manifestations of this process occur during embryonic development, regeneration, and metamorphosis; smaller-scale, but equally important, is the on-going maintenance of order that can resist aging, degradation, and cancer over decades [[1], [2], [3], [4]]. It's a remarkable process in which large numbers of cells cooperate to reliably build, and remodel toward an invariant target morphology.

A key aspect of morphogenesis is that it is not an open-loop (purely feed-forward) process [5]. It is often assumed that the conceptual tools of emergence and complexity science can capture this process: local rules executed recursively in parallel across all cells, will result in the generation of complex growth and form. This is certainly true; indeed there are now many computational models of complex patterns emerging from simple processes [[6], [7], [8], [9], [10]]. However, there is an additional and crucial component of biological morphogenesis which is missed by purely emergent models: anatomical homeostasis (or more accurately, homeodynamics), in which the system can pursue multiple different paths in order to achieve the same anatomical outcome despite novel circumstances. In effect, biological morphogenesis is not well-described by bottom-up, open-loop, mechanical models [[11], [12], [13]], but instead exhibits more complex policies as it navigates the space of possible anatomical shapes despite various perturbations [14]. This includes the ability to reach the correct target morphology despite damage (e.g., bisection of early embryos), scrambling of tissue positions, changes of cell size and number, as well as numerous alterations of the genome (reviewed in Refs. [5,[14], [15], [16], [17]]). Numerous kinds of biochemical, biomechanical, and bioelectric prepatterns have been described which can encode setpoints for anatomical homeostasis [[18], [19], [20], [21], [22], [23]].

Understanding how bodies navigate anatomical morphospace, including the nature and limits of reliability, the possible failure modes, and the optimal intervention strategies is crucial for advances in evolutionary developmental biology, the bioengineering of synthetic life forms, the biomedicine of birth defects and regenerative therapeutics, and adaptive robotics [24,25]. This specifically requires models of how the behaviors of individual cells scale up to adaptive responses at the level of the entire body. What mechanisms allow the coordination of cell activity toward one consistent morphology? In particular, any theory has to provide a hypothesis for how the homeostatic capabilities of individual cells relate to the much more complex homeostatic setpoint of the collective.

One candidate mechanism that has been proposed is stress – a concept with a rich history in physics and materials engineering and a good candidate for a generic principle of biological organization at the system level [[26], [27], [28], [29]]. Stress is a popular topic in biology, studied at the level of DNA damage and protein misfolding, tissue morphomechanics, immunomodulation, behavior, and even whole ecosystems [27,[30], [31], [32], [33], [34], [35], [36], [37]]. We have previously proposed [38] that the generic concept of stress – as a biophysical marker of the computed error (distance between the current state and a homeostatic setpoint) – can be extended to the morphogenetic process. For example, the limb of the salamander will re-grow when amputated; the most remarkable part of this process is that it knows when to stop: growth and remodeling ceases when a correct salamander limb is completed [39,40]. Similarly, when a tail is transplanted to a limb-specific location in amphibia, it slowly remodels into a limb – the structure more appropriate to the bodyplan [41,42]. This, and other examples such as craniofacial corrections of scrambled amphibian faces [[43], [44], [45]], can be modeled as a progressive error minimization: cells continue to move and adjust until the error is within tolerance. The impetus that maintains activity could be seen as a stress reduction drive, as occurs at every other level of biological organization. Thus, in our models, stress reflects the degree of error – the distance (in this case, in anatomical morphospace) that the system can detect with respect to its prepattern. It is clear that many biological systems can detect and correct anatomical error (see examples in Refs. [5,17]); here, we focus not on these mechanisms but on the dynamics of the mediator of error information: stress.

**Fig. 1:**
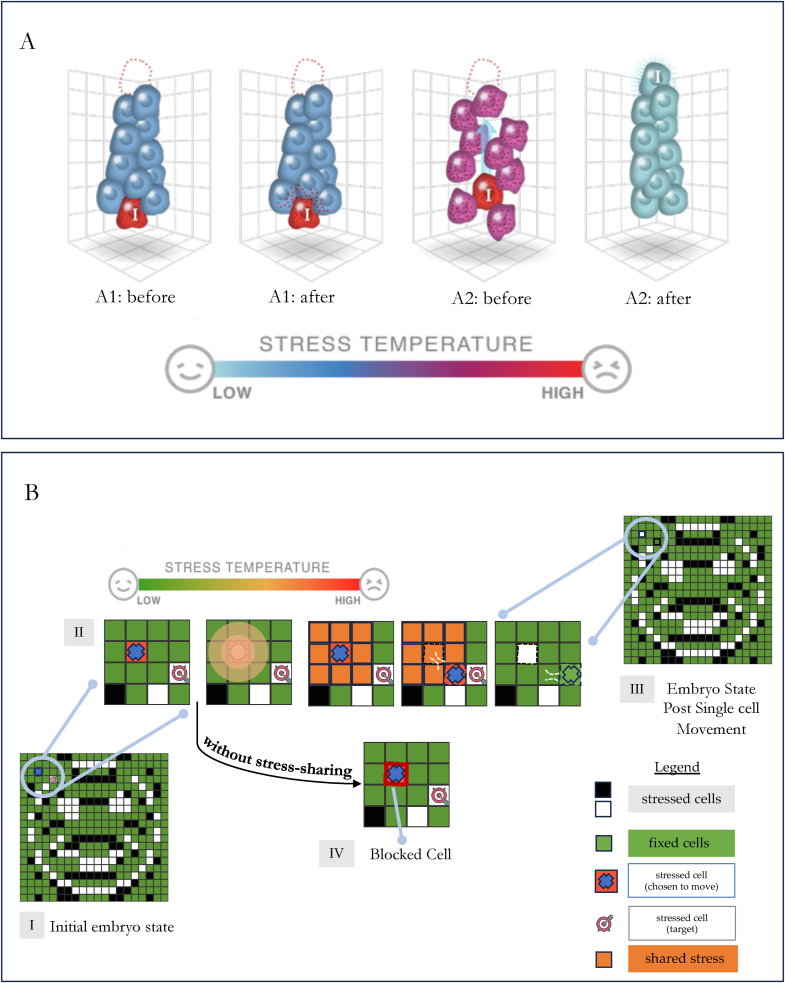
Schematic of experimental setup (A): A simplified diagram of one typical scenario during morphogenesis in which stress sharing is hypothesized to help solve the problem. Cell *I* (red) is motivated to move upward until it reaches a specific position in a morphogen gradient. (A1) Before and after, in the absence of stress sharing: cell *I* will not get to its intended spot because all the other cells' target states are met and they have no incentive to move enough to let cell *I* pass, no matter how much stress cell *I* is feeling due to the error state of its current position. Thus, a permanent defect will remain. (A2) Before and after, in the presence of stress sharing: if the stress molecule(s) representing the delta between its gradient setpoint and its current position can leak (or be exported) our of cell *I*, they will affect nearby cells. Since stress molecules are conserved among all cells within a given body, the neighboring cells will be stressed by their microenvironment, which raises their plasticity and enables them to let cell *I* pass through. When it gets to the correct position, its stress levels reduce and the whole cell sheet's stress levels reach low levels because the entire collective is in a low-energy state with respect to all of their setpoints. In this way, a systemic stress response mechanism, implemented by conserved stress molecules that leak from specific cells, could facilitate large-scale problem-solving by de-localizing incentive to work toward group goals (cooperation without built-in altruism, since all the cells are simply trying to reduce their own local stress levels). The leak mechanism enables the collective to bend the energy landscape for the individual cells to exploit their homeostatic optimization behavior towards a tissue-level patterning goal. (B): Process of cell movement with and without stress sharing in embryos: The target task was for cells within a randomly initialized 2D matrix/embryo to re-arrange themselves towards a pre-set target pattern (a smiling face binary image in our setup). Stressed cells (B.I) (i.e, cells which are not in their correct positions to form the pre-set morphogenetic pattern) were allowed to move by picking other stressed cells as target and moving towards them through local swaps. By default, a stressed cell was not allowed to disturb other fixed cells (cells which were already in their correct positions). However, when given the ability, stressed cells could share their stress with their fixed neighbors, urging them to provide a path for movement towards the target (B.II). Consequently two kinds of competencies were setup: One with stress sharing (B.II), and one without (B.IV). In the case where sharing was not allowed, a stressed cell surrounded by other fixed cells would have no way of moving through, causing it to get blocked locally (B.IV). In the case where sharing was allowed, blocked stressed cells would spread their stress to an area of size 3x3 around them (shown in orange), thereby communicating an intent of movement; in response, a channel would be created by the fixed cells for the stressed cell to move towards the target location. (Stages I, II, and III). With each resolved cell movement, the stress map was updated (B.III). Such a process occurred iteratively until either the competency limit was reached or the target pattern was formed. (For interpretation of the references to colour in this figure legend, the reader is referred to the Web version of this article.)

While the molecular mechanisms of stress sensing, and the degree of conservation of specific molecules as bearers of the systemic stress signal from subcellular to multicellular scales, are currently under study and largely unknown, one interesting hypothesis concerns the role of stress *sharing* as a mechanism for the functional cooperation of cells in vivo. Consider a cell in the wrong position of a developing tissue and its positional information gradient (cell “I”, in Fig. 1A). The cell is motivated to move, to relieve its stress; however, its neighbors are at their correct positions and their low stress causes strong functional inertia not to move away from their current low-energy configuration. The individual cell-scale homeostatic loops prevent cooperation in this case and the optimal anatomical configuration is not reached (Fig. 1.A1).

However, one simple way to resolve this problem would be to allow stress sharing molecules to leak out of the source cell and diffuse outward to neighboring cells. If all cells interpret these conserved, shared molecular signals as stress, a given cell cannot tell if its high stress sensation is due to a problem *it* has or a problem *its neighbors* have. A cell that is leaking stress raises the exploratory activity level temperature of the nearby cells [46] (a quantity known as temperature, in the physics of annealing systems), making them more plastic and willing to perform active behaviors because they do not rate their current state as a satisfactory, low-energy stable state. This lowers the barrier for them to undergo exploratory motion in the space, allowing the other cell to move through to lower its stress level, at which point the whole tissue is at the optimal lowest-energy configuration and every cells' stress is reduced (Fig. 1A2). This kind of scheme is common in computational models of biological networks [[47], [48], [49]], and produces cooperation without explicit mechanisms of altruism. By sharing stress with neighbors, one cell's (or region's) problem becomes *everyone's problem*, leading to a higher willingness to adopt new configurations in which everyone's free energy is lower (and thus, more optimal morphogenetic problem-solving).

This hypothesis makes some strong predictions about the robustness of morphogenesis with and without such a stress sharing mechanism. However, in multiscale systems with emergent behaviors it is often not obvious how well such a mechanism would work, or what the resulting dynamics would be, without quantitative simulation. Here, we test those predictions in an *in silico* model of morphogenesis. In our model, migrating cells working to implement homeostatic target morphologies were either allowed or disallowed from sharing their stress during the process of rearranging in space.

We analyzed this model in three conditions, with the first being designed to test the impact of stress sharing and the last two being null hypothesis controls. First, our experimental mode with stress sharing, in which the individual cellular homeostats, in which stress is proportional to distance from their homeostatic setpoint, do propagate their stress to other cells. By analyzing these results, we find interesting phenomena that suggest that stress sharing is an easy way to achieve robustness in collectives made up of homeostatic subunits.

Second, a mode in which cells are agents and possess certain competencies that modify the resulting phenotypic fitness – they still have homeostatic loops governed by stress but they do not export it to their neighbors [50,51].

Third, a “direct development” mode in which phenotypes are hardwired from the genotype, mirroring many uses of genetic algorithms and numerous theoretical biology or artificial life studies which omit the developmental layer between them (no homeostatic competency or regulative development). By comparing the data, we find interesting and important roles of stress sharing as a dynamic that facilitates collective problem-solving.

## 2 Methods

We modeled (see Supplements S1 and S2 for technical approach and code execution details) embryos achieving a target morphology in-silico; the target morphology we chose was similar to the “electric face” bioelectric prepattern known to exist during frog embryogenesis [52]. We assessed the impact of embryogenesis on the morphogenetic process by encoding embryos with different developmental routines and comparing their reorganization potential. All developmental routines involved varying degrees of stress sharing between cellular components as encoded by their genome. Apart from comparing developmental routines on an individual scale, we also sought to compare its effects on an evolutionary scale by wrapping the developmental process within an evolutionary algorithm.

In our framework, an embryo was treated as a collection of cells within a two-dimensional (2D) grid. We chose a two-dimensional (2D) matrix data structure to be its computational equivalent for modelling, referring to its elements as cells (analogous to biological cells). Thus, our biological tissue is simplified as a discretized lattice, conceptually related to Chua's cellular neural networks [53]. Embryos were grouped into three different kinds based on their cells' ability to reorganize themselves during development: (1) stress-sharing embryo: wherein cells moved by sharing their stress, (2) without-stress-sharing embryo: where cells could move, but could not share their stress levels with others, and (3) hardwired: where cells could not move at all. The first two types thus included a developmental layer between genotype and phenotype, while the third simulated an organism with no regulative development, where the connections between genotype and phenotype was much more direct [51].

**Fig. 2:**
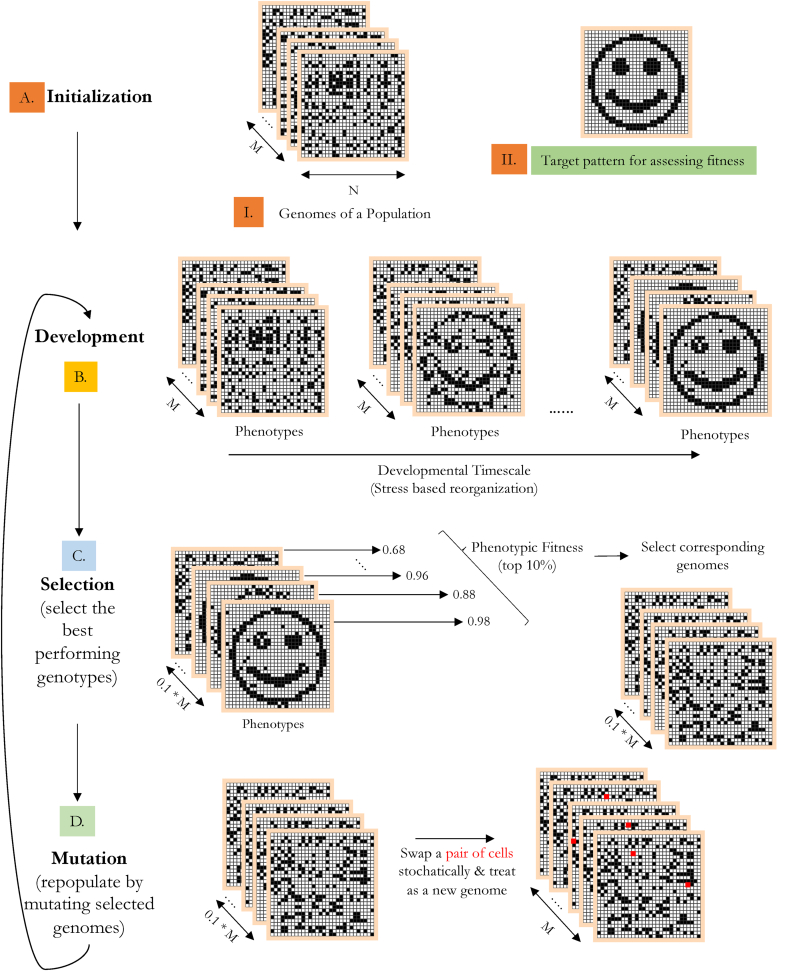
Genetic algorithm Schematic (A): Initialization: Prior to evolution, a population of genomes which served as the substrate for evolution were initialized. A genome in our framework was a randomly initialized two-dimensional matrix containing two cell-types (0, indicated in black, and 1, indicated as white) (A.I). The genome also carried a marker indicating the type of development an embryo could undergo during the first stage of evolution i.e: development. Three kinds of reorganizaton processs were defined: 1. Stress sharing based reorganization, 2. Reorganization without stress sharing, and 3. Hardwired (no reorganization). A target for evolution was also set. To this end, we chose a downsampled binary image of a smiling face as the target pattern which evolution had to optimize over (A.II). (B): Development: The first stage of evolution involved a process of cellular reorganization of the initialized matrices. Reorganization would be based on the gene marker encoded in the genomes of each embryo. This stage served to mimic the developmental stage observed during an organism's lifetime. Post development, the reorganized cell-structure was termed the embryo's phenotype. Thus, the developmental process served as an independent layer separating the genotype of an embryo from its phenotype. (C): Selection: Prior to development, the fitness of each genome was recorded by comparing it with the target (l2−distance). And Post development, the fitness of the corresponding phenotype was also recorded using its l2−distance with the target. During selection, phenotypic fitnesses of the top 10 % of the population were chosen and, their corresponding genomes were selected (Darwinian selection) for evolution in the next generation and the rest were discarded. (D): Mutation: The selected for genomes constituted 10 % of the original population. To repopulate the population back to its original strength, selected genomes were mutated by randomly swapping cell-pairs within the genome. The resulting mutated genome served as a new embryo. Post re-population, a new cycle of evolution began marked by the onset of development.

Our primary goal was to observe how the presence of a developmental process, -- which acted as a layer separating the initial substrate of the embryo from its reorganized state -- altered the evolutionary dynamics of a population. To quantify this, we evolved populations of embryos using a genetic algorithm (GA). Since our genetic algorithm had to account for a developmental stage during the life cycle of an embryo, it was an iterative sequence of the following stages: 1. Development, 2. Selection, and 3. Mutation (Fig. 2).

During initialization, each embryo (i.e., each 2D matrix) was initialized with one of two cell types (0 or 1) and were patterned in a stochastic manner to give a scrambled appearance to their structure (Fig. 2A.I). Each embryo within a population was initialized similarly with a unique scrambled pattern and the specific pattern itself was treated as its genome. In addition, the genome also encoded a separate value (the reorganization gene marker) which indicated the type of developmental reorganization it could undergo during the upcoming developmental stage.

### 2.1 Stress-based development

Post initialization, a population of embryos underwent development (the first stage of the genetic algorithm). During development, embryos reorganized their cells to resemble a target two-dimensional pattern which we pre-specified in our experiments to be binary image of a smiling face (Fig. 2A.II). Based on the kind of reorganization gene marker encoded in its genome, an embryo could either undergo reorganization by sharing its stress, by not sharing its stress, or by not using stress at all.

Stress was a binary signal which indicated whether a particular cell was in its correct x,y coordinate position (with respect to the target) or not. Since our initial embryo was scrambled, most of its cells were bound to be stressed. Stressed cells were inherently driven to move somewhere (they possessed a sense of restlessness), the question of where to move to was based on another signal which we called the distress signal.

A distress signal originated in those cells which were *not* stressed (we called these “fixed cells”). Each of these fixed cells had a tendency to observe their neighbors (a 3x3 neighborhood around them) and to check if there were any stressed cells present. On recognizing the presence of a stressed cell in a particular direction within their 3x3 neighborhood, they would send a radially diminishing signal (which had a strength of 1.0 at its origin, but radially diminished in strength) encoding the presence and the location of a stressed cell. This process was analogous to how cells at a wounded site send signals elsewhere requesting assistance. In our case, a fixed cell, requested assistance in a particular location by sending a distress signal.

It is important to note that not all cells received this distress signal. First, only stressed cells received it, and second, only those stressed cells carrying a cell-value of the opposite kind received it: As an example, suppose a fixed cell (say, F1) identifies a stressed cell (say, S1) of cell-type “0” located on top of it; the stressed cell, S1, is precisely stressed because it is of the wrong cell type at the wrong x,y location). Thus, the fixed cell, F1 recognizes this and sends a distress signal to the rest of the 2D grid, requesting some other distant stressed cell (say S2) with the opposite cell-type “1” to move to the place of S1, thereby relieving its stress.

The above example is a simplification; in practice, multiple distress signals were sent out simultaneously, and stressed cells could receive and accumulate these signals to move to a specific target location. Once distress signals were captured by stressed cells, they moved.

Movement occurred sequentially. At each instance, a single stressed cell in the grid was chosen at random, and its distress-signal-record from various sources was consulted. Its destination for movement was chosen as that location from which the distress signal was strongest in value. The chosen stressed cell moved to its target by repeatedly swapping with intermediate elements in the direction of shortest path.

### 2.2 Types of cell-movement (cellular competency) and the concept of stress-sharing

During cell movement, we had to deal with a problem: what would happen to the fixed elements which were in the way of a stressed cell as it moved towards its destination? If the stressed element swapped with fixed cells, it would dislodge them causing them to become stressed (thereby increasing the overall stress rather than decreasing it). We solved this problem by disallowing stressed elements to swap with intermediate fixed elements i.e., by default, if a stressed element encountered a fixed element while making its way towards its chosen destination, it could not move any further and would get stuck.

A special case which over-rode this default, was when we pre-specified stressed cells to share their stress with its neighbors. Since stress was a binary signal (0 or 1), a stressed cell communicated its status as a stressed cell to a fixed neighbor during movement. This communication process was termed “stress-sharing” and the effect it had was a coercion of the fixed cell to create a passage (a de facto tunnel) for movement through it rather than by swapping with it (Fig. 1B). Thus, on receiving a shared signal of stress from a stressed cell moving towards its destination, the fixed cell created a temporary channel, which allowed the stressed cell to swap with other stressed cells through it. In case multiple fixed cells stood in the way of the moving stressed cell, stress sharing ensured a tunnel was created through all of them for movement.

The tunnel induced as a result of the shared stress was temporary. Once the stressed cell moved away from the tunneled fixed cell, it reverted back to its original “blocking state”. Such a stress sharing mechanism, allowed the possibility of free, unrestricted movement towards all parts of the discrete grid, preventing dislocation of fixed cells from their grid positions during cell-movement. An alternate form of the reorganization process, one which did not involve stress sharing was also considered. In such cases, a stressed cell would get stuck as soon as it encountered a fixed cell while moving towards its destination in the direction of shortest path.

To summarize, an embryo could undergo one of three different kinds of reorganization: 1. With stress sharing, 2. Without stress sharing, and 3. Hardwired. Since the first two methods involved local cell movement they were termed competent strategies. The hardwired case served as a control to the other two competent strategies. The cells of a hardwired embryo could not move and thus lacked a reorganization process; development during evolution only served as a placeholder, without any functional significance.

Post-development, embryos possessed a transformed structure and the resulting grid organization was termed its Phenotype. Thus, the developmental stage served as an independent layer separating the genotype from the phenotype. Definitions of the genotype and the phenotype, as they have been used here, have loose connections with biology and assume special meaning only here. Demarcating these two phases allowed us to monitor the impact of the reorganization process on fitness throughout evolution (Fig. 2).

### 2.3 Fitness function

Our genetic algorithm served as a search process over genotypes which best reorganized themselves according to the target pattern (a smiling face in our experiments). The fitness value of any embryo was determined by its l2distance, d with the target.

$$d=\frac{1}{N^{2}}\;{\left(\sum_{i=0}^{N}\sum_{j=0}^{N}{\left(E_{i,j}-T_{i,j}\right)}^{2}\;\right)}^{\frac{1}{2}}$$

Where $E_{i,j}$ and $T_{i,j}$ are the (i,j)th elements of the embryo and the target respectively. Intuitively, the fitness function provided a quantitative metric of how close the embryo was to the target morphogenetic pattern.

During evolution, we noticed significant differences between populations in higher fitness values than lower fitness values. To make this apparent, we zoomed into higher fitness values by exponentiating the l2distance as follows:

$$d^{\prime }=\frac{9^{d}}{9}$$

Given the presence of a developmental step, two separate configurations of the embryo existed during a single cycle of the GA: 1. The state of the embryo prior to development (as the genotype) and 2. The state of the embryo post development (as the phenotype). Consequently, two kinds of fitness existed: the genotypic fitness, which measured the fitness of the genotype; and the phenotypic fitness, which measured the fitness of the phenotype. An exception occurred in hardwired embryos: since they lacked competency, their phenotype was equivalent to their genotype and thus their phenotypic fitness was equivalent to its genotypic fitness.

### 2.4 Kinds of embryos

The genotype of each embryo carried a “reorganization-marker” indicating the kind of developmental reorganization it could undergo during development. Each embryo was categorized into three different kinds based on this gene marker as follows: 1. With stress sharing embryo. 2. Without stress sharing embryo, and 3. Hardwired embryo. The first two categories had a general ability for reorganization, and were termed as “competent”, in the sense of problem-solving ability of active tissues to restore correct shapes despite induced barriers, perturbations, and other interventions [5,54]. Alternatively, the hardwired embryo was not-competent and possessed a fixed structure throughout development.

### 2.5 Populations for evolution

Our genetic algorithm evolved populations of embryos of three different kinds: 1. Stress sharing population, 2. Without stress-sharing population, and 3. Hardwired population. To create a population of size M; M 2D-matrices of size N were initialized by randomly scrambling the target pattern (also of size N). We initialized grids this way in order to maintain the same number of cell types (0’s and 1’s) across both the initialized embryo and the target. It ensured simplicity and allowed us to focus on reorganization rather than “growing” the required kind of cell types to satisfy the target pattern.

### 2.6 Genetic algorithm

We evolved each population by passing it iteratively through a sequential process of: 1. Development, 2. Selection, and 3. Mutation. During the selection stage, phenotypic fitnesses were used to select the best genotypes for the subsequent generation (Darwinian selection). In order to repopulate the population with variant genotypes we mutated the selected genotypes by stochastically moving a few cells to random locations. At the end of each generation, we plot the phenotypic fitness and the genotypic fitness of the embryo with the highest phenotypic fitness in the population (see Supplement S2 for more details).

### 2.7 Competency value and cell-Distance

Embryos with competency (either with stress sharing or without stress sharing) were given a pre-determined “competency value”. This value provided an upper bound to the number of swaps the cells of the embryo could execute during the developmental step. We also monitored the total distance travelled by these cells by calculating the average Euclidean-distance moved by elements during a generation.

### 2.8 Statistical analyses

To observe the impact of stress sharing during evolution, we plot the fitness curves of each population (with stress sharing, without stress sharing, and hardwired), over 1000 generations. 95 % confidence intervals (CI) over 10 runs of each experiment were plotted to verify if any of the CI bands of any population overlapped with each other across 1000 generations. A lack of overlap suggested a significant difference in evolutionary dynamics. Further, we measured the statistical difference between different populations at arbitrary points during evolution and carried out a *t*-test between samples of any two populations to quantify their similarity.

## 3 Results

### 3.1 Stress sharing helps populations solve the problem of morphogenesis faster

To determine whether a cellular rearrangement process involving stress sharing helps in discovering the optimal morphogenetic process faster, we compared the evolution of three different populations in-silico, each with a different kind of cellular rearrangement process over 1000 generations.

**Fig. 3:**
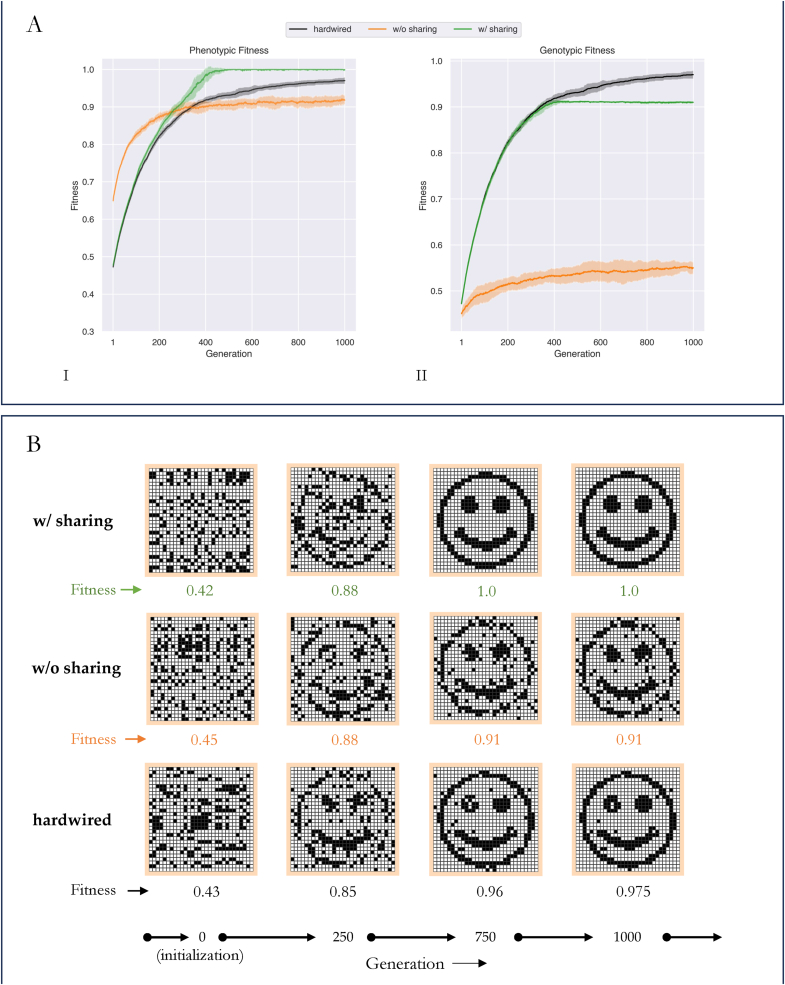
Stress Sharing results in solving the problem of morphogenesis faster than without stress sharing or without reorganization competency (hardwired) (A).I: Phenotypic fitness curves of populations of embryos (size 30x30) post competent cellular rearrangement are shown. Populations with stress sharing based competency (green) reached maximum fitness by generation 400, which was faster than populations without stress sharing (orange) (p≪0.01) or those without any competency at all (black curve; hardwired population) (p≪0.01) at generation 400. In the absence of stress sharing (orange), a competent population performed worse than a hardwired population (black) post generation 400. (A.II): Genotypic fitness curves of populations of embryos (size 30x30) prior to competent cellular rearrangement. The genomes of a competent population w/stress sharing improved in a manner similar to that of a hardwired population until ∼ generation 400 (p=0.9), post which it plateaued. (B): Embryos post competent cellular rearrangement (phenotypes) at different stages of evolution are shown. Grids correspond to those embryos with the best phenotypic fitness in any generation. (B.I) (w/sharing): Starting from a randomly initialized grid of binary values (generation 0, row1, column 1), the target pattern (a smiling face) was achieved by ∼ generation 400 (Fig: A.I) (B.II) (w/o sharing): Similar to row 1, embryos were initialized randomly with a different seed value. By generation 1000 (row 2, column 4), the final pattern was partially achieved. (B.III) (hardwired): In case of a hardwired embryo, its cells were not allowed to move. Thus, improvements were possible only through mutations to the grid structure. We noticed that the target pattern was almost achieved in this case, outperforming a population w/o sharing (row3) (fitness of 0.975 (hardwired) vs 0.91 (w/o sharing) at generation 1000). (For interpretation of the references to colour in this figure legend, the reader is referred to the Web version of this article.)

The experiment consisted of running a population of embryos through a genetic algorithm. The genetic algorithm (GA) involved an iterative process of development, selection, and mutation. The developmental step was introduced to allow a genotype to change into a phenotype before selection (Fig. 2). Three different populations were evolved through our GA: 1. a population which relied on stress sharing to rearrange itself; 2. a population which relied on stress but had no sharing capacity, 3. a population which had no rearrangement capability (hardwired). We plot the phenotypic fitness (Fig. 3A.I) and the genotypic fitness (Fig. 3A.II) of the embryo with the highest phenotypic fitness in each of these populations over 1000 generations averaged over 10 runs to observe their morphogenetic ability.

We observed that populations with stress-sharing were able to discover the correct genotype and the competency necessary to form the target pattern faster than the other two populations (Fig. 3A). Members of this population discovered how to arrive at the target pattern by generation 500, at which a population with sharing had a significantly different phenotypic fitness from either the hardwired (p≪0.01), or that of the population without sharing (p≪0.01). Further, the confidence intervals of these curves did not overlap, indicating their distinct separation from one another (Fig. 3A.I).

The way in which stress sharing populations derived their advantage was by progressively improving their genotype during the early stages of evolution (Fig. 3A.II), behaving as if they were a hardwired population (we found no statistical difference between the two until generation ∼400 (p=0.9). In contrast, populations without stress sharing improved their genomes only fractionally above their initialized states (Fig. 3A.II) and relied exclusively on their competency to achieve high phenotypic fitness towards the target (Fig. 3A.I). Such a strategy paid off early during evolution (until generation ∼400) but resulted in diminishing gains post this point (Fig. 3A.I).

We verified the phenotypes of the best embryo in each of these populations at generation 0, 250, 750, and 1000. We observed that at generation 250, all three populations had a similarly distorted pattern as confirmed by their approximately similar fitness values (Fig. 3B). Further, the stress-sharing and the without stress-sharing populations had a similar fitness value (=0.88) at this point (Fig. 3A.I, p=0.97), even though the population without-sharing engaged in a different dynamic compared to the other two populations (Fig. 3). At generation 750, despite their similar dynamics early on during evolution, the embryos with stress sharing were capable of forming the target pattern, whereas the hardwired population could not; however, we noted that it does perform better than the population without-stress sharing. At generation 1000, we observed the hardwired embryos being closer to maximum fitness with a positive slope of 0.19 compared to the population without sharing (slope = 0.05).

We conclude that populations with stress sharing discover the correct morphological solution faster than hardwired populations or populations without stress sharing. Hardwired populations, relying on mutations alone, are eventually capable of achieving high fitness, while populations without-sharing are worse than hardwired populations over long time scales.

### 3.2 Stress sharing helps populations with a range of complexity solve morphogenesis

**Fig. 4:**
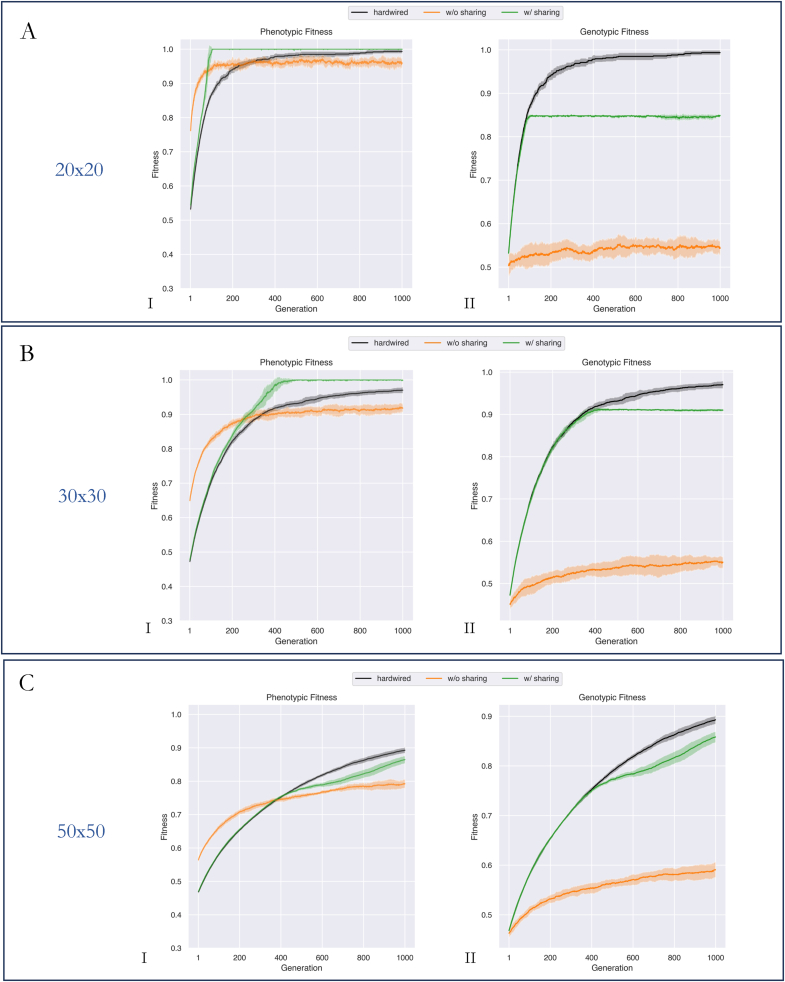
As grid size increases, the morphogenetic task grows harder; a competent stress sharing mechanism helps in navigating a population to the target solution (A): Fitness graphs (phenotypic and genotypic) for populations with embryo sizes of 20x20. A competent population w/stress sharing solved the problem by generation 100 (A.I), whereas both the hardwired as well as a population w/o sharing were unable to achieve perfect fitness by generation 1000. Further, the hardwired population performed comparatively better than a population w/o sharing post ∼ generation 350 (p≪0.01). Genomes of the w/sharing population evolved identically to that of a hardwired population until ∼ generation 100 (p = 0.96) post which we observed a plateauing of its genome quality (A.II). (B): Fitness graphs for populations with a grid size of 30x30. A population w/sharing solved the problem in ∼400 generations, whereas the hardwired population as well as the population w/o stress sharing, failed to reach maximum fitness by generation 1000, obtaining a fitness of 0.975 and 0.91 respectively (p≪0.01) (B.I). Genomes of the stress-sharing and hardwired populations had similar early stage dynamics (∼generation 400, p = 0.96), post which the stress-sharing population's genome stagnated. The without-sharing population's genome improved by less than 20 % above its initialized state (4B.II). Further, we observed that these dynamics were all right-shifted versions (with respect to time) of the experiment with grid size 20x20. Generally, populations of size 30x30 took a longer time to reach the same phenotypic fitness level as in 20x20, and genomes of the stress-sharing population stabilized after a longer time (∼300 generations longer). (C): Fitness graphs for populations with embryo sizes of 50x50. A competent population w/sharing (green) improved identically (in phenotypic fitness) to a hardwired population (black) until generation 400 (p=0.95), by generation 1000 we noticed that its performance was climbing back up to hardwired levels (C.I). Genomes of the w/sharing population improved identically to that of the hardwired population until generation 400 (p = 0.98). Post which a deviation (identical to that observed in the phenotypic fitness curves) were noticed. By generation 1000, genomes of the w/sharing population were improving to the level of hardwired individuals (C.II). We were unable to run this experiment for longer timescales owing to resource constraints. However, we note that both the genotypic and phenotypic dynamics at this scale are right shifted (in-time) versions of lower grid sizes (20x20, and 30x30). Consequently, we expect similar future trends in their phenotypic and genotypic fitness. (For interpretation of the references to colour in this figure legend, the reader is referred to the Web version of this article.)

In our first experiment, each embryo within a population was a matrix of size 30x30. To determine how sharing benefits populations of cells of different sizes, we initialized multiple instances of the previous experiment each with a different grid size and compared their fitness curves over 1000 generations. Specifically, we ran three instances of the previous experiment, with grid sizes 20x20, 30x30, and 50x50. Within each instance, we compared the performance of the stress-sharing population with the hardwired and without-sharing populations by observing their fitness curves (Fig. 4).

We observed that for a grid size of 20x20 (Fig. 4A), stress-sharing populations were able to reach maximum phenotypic fitness by ∼ generation 100; hardwired populations were able to reach maximum phenotypic fitness at generation 1000 and a population without sharing was unable to reach phenotypic maximum fitness even after 1000 generations of evolution (Fig. 4A.I). The genomes of the stress-sharing and hardwired populations evolved identically during the early stages of evolution (until ∼ generation 100 throughout which we noticed no statistical difference (p=0.96) post which the stress-sharing population stabilized. (Fig. 4A.II). Genomes of the without-stress-sharing population improved marginally above its initialized state.

At a grid size of 30x30, a similar pattern of evolutionary behavior emerged: the stress-sharing population reached maximum phenotypic fitness faster, followed by hardwired with a rising slope (=0.19) at generation 1000, and the without sharing population with a stable phenotypic fitness around ∼0.91 at generation 1000 (Fig. 4B.I). Genomes exhibited identical patterns as well: the stress-sharing and hardwired populations had similar early stage dynamics (until generation 400) (p=0.96), post which the stress-sharing population's genome stagnated. The without-sharing population's genome improved by less than 20 % above its initialized state (Fig. 4B.II). Further, we observed that these dynamics were all right-shifted versions (with respect to time) of the experiment with grid size 20x20. Generally, populations of size 30x30 took a longer time to reach the same phenotypic fitness level as in 20x20, and genomes of the stress-sharing population stabilized after a longer time (∼300 generations longer).

At a grid size of 50x50, 1000 generations were insufficient for any of the three populations to reach maximum phenotypic fitness (Fig. 4C.I). However, general patterns similar to those seen at lower grid sizes were observed: phenotypic fitness of the stress-sharing population evolved similarly to the hardwired population until generation 400 (p=0.96), followed by a short term decrease, before rising back up to hardwired levels. The without-sharing population exhibited early gains but stabilized closer to generation 1000 (Fig. 4C.I). Trends in genotypic fitness revealed a similar dynamic to those seen in lower grid sizes (20x20, and 30x30): the hardwired and stress-sharing population evolved similarly, however, unlike lower grid sizes, its dynamics dropped momentarily before reaching hardwired levels. The without-stress sharing population exhibited relatively modest improvement of its genome, but we note that it is higher than either of the previous two grid sizes.

We conclude that as grid size increases, the problem of morphogenesis grows harder, with populations requiring a longer time to reach peak fitness. Despite the increase in complexity, a stress-sharing population can utilize its competency to reach maximum fitness. The benefit of stress-sharing is especially noticed in the later stages of evolution when mutations cannot be relied upon to solve the problem efficiently, and more precise cell movements are required.

### 3.3 Stress sharing encourages cell-movement over long distances

How does stress sharing help the collective achieve its morphogenetic objective? We next tested the hypothesis that the sharing of stress information facilitates the movement of cells over longer distances. To quantify its effect to fitness improvement, we plotted the competency value and the average Euclidean distance travelled by cells of the best embryo in each of the three populations throughout evolution.

**Fig. 5:**
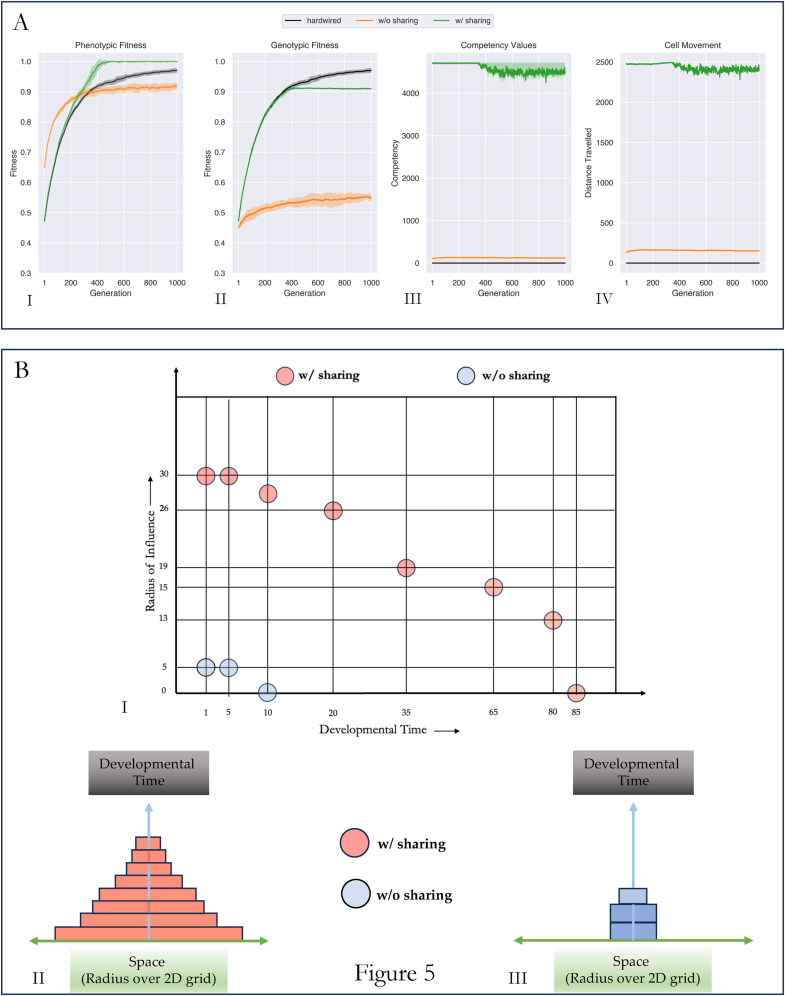
Stress sharing is beneficial because it encourages cell movement over long distances. (A): Evolution of a population of embryos each with a grid-size of 30x30 over 1000 generations. Genotypic fitness of an embryo with the best phenotypic fitness (A.I), its corresponding phenotypic fitness (A.II), its competency value (A.III), and the average distance travelled by its cells (A.IV) at each generation is shown. A population w/o sharing increases its phenotypic fitness rapidly in the first 200 generations during which it utilizes a competency of 100 units and its cells move an average of 250 units. Post generation 200, its phenotypic fitness stabilizes owing to its low genotypic fitness and constant utilized competency. Populations with stress sharing behave as if they were hardwired for the first 400 generations despite utilizing a high degree of competency (∼4725 units) (A.III), post which they reach maximum fitness and reduce their competency usage by ∼150 units. A high utilization of competency consequently allows cell movement across large distances in the matrix. Greater freedom causes cells of a stress sharing population to move as if they were random during the first 400 generations, post which their freedom enables them to achieve cell states with high fitness. (B): Stress sharing increases the radius over which a cell influences another. (B.I):Two embryos of size 30x30, one with stress sharing and one without are developed independently. At each stage, the maximum distance over which a cell influences another cell by changing its future fate is recorded and plot at different intervals in time. As a cell moves towards a distant target, it dislodges other cells lying in its path, effectively changing their future fates. The radius of influence serves to measure the maximum distance of such influence. The embryo with sharing has a radius of influence of 30 at developmental stage 1, and decreases in a non-linear manner, eventually reducing to zero at stage 85. On the other hand, an embryo without sharing has a radius of influence of 5 at stage 1 which terminates to zero at stage 10. (B.II, B.III): An alternate view of the radius of influence over time for an embryo with sharing and an embryo without sharing respectively. Rectangular blocks of width proportional to the radius of influence are stacked on top of each other. Blocks are representative of the radius of concern at developmental stages marked on the x-axis. An embryo with sharing exerts influence over a larger radius for a longer duration of developmental time (5.B.II) than an embryo without stress sharing (5.B.III).

Specifically, we initialized populations with members of size 30x30 and ran them through our GA for 1000 generations. In each generation, we picked the embryo whose phenotypic fitness was the highest and traced its genotypic fitness (Fig. 5A.II), competency value (Fig. 5A.III), and the average Euclidean distance travelled by its cells within that generation (Fig. 5A.IV). Each embryo was given a maximum possible competency value of 4725 which was determined as a function of its grid size, N (see Supplement S3)

We observed that the stress-sharing population utilized maximum competency (=4725) until ∼ generation 400, beyond which its value dropped to ∼4500 and remained approximately stable thereafter. The population without stress-sharing utilized a competency of ∼100 throughout evolution and the hardwired population used 0 competency because it wasn't allowed to use competency by design (Fig. 5A.III).

Further, the cells of the best embryo (i.e., with the highest phenotypic fitness) in the stress-sharing population moved an average Euclidean distance of ∼2500 units until ∼ generation 400, post which it varied around a value of ∼2400 units. In case of the population without stress-sharing - owing to its lower usage of competency – cells moved an average of ∼200 units throughout evolution (Fig. 5A.IV).

We conclude from these observations that stress-sharing is beneficial precisely because it enables cells to move over longer distances through a stress-communication mechanism (Fig. 5A). Owing to the approximate nature of such movement, stress-sharing embryos are capable of utilizing a high degree of competency (Fig. 5A.III) to solve the morphogenetic puzzle. In contrast, a population without stress-sharing lacks such a communication mechanism and is therefore restricted to local cell-movements. Movement restriction enforces a limit on competency causing embryos to perform poorly. The Hardwired population leverages mutations to solve the morphogenetic task better than a competent population with no stress sharing.

### 3.4 Stress sharing increases cells’ radius of influence

We then sought to study these dynamics from the perspective of the cognitive light cone model [55], which focuses on the radius in space and time of events that any given agent can use as a homeostatic setpoint. In effect, taking the agent's perspective in terms of what it functionally “cares about” [[56], [57], [58], [59]].

As a cell moves towards its target position during development, it disturbs other cells, knocking them out of position and altering their future fates towards a target position. To quantify the range over which a given cell alters the fate of another cell, we plot the maximum radius over which a cell dislodges another cell at each stage of development. Specifically, we took a single embryo of each kind (with sharing and without sharing), passed it through the developmental stage, and plot the maximum radius over which cells dislodged other cells in each developmental step (Fig. 5 B).

We observed that in an embryo with stress-sharing, a cell influenced another cell located a radius of 30 units on average at developmental step-1. This distance progressively decreased over development, with the value dropping to zero at step 85 (Fig. 5B.I). In contrast, in an embryo without sharing, a cell altered the fate of another cell located at a radius of only 5 units on average at development step 1, with development ceasing completely by step 10. (Fig. 5B.I).

An alternative view of the radius of influence over developmental time is shown in Fig. 5 B.II, and 5.B.III, which better illustrates the impact of stress-sharing over developmental time. We plotted the radius of influence as a rectangular bar of proportionate size, and stacked multiple such blocks at developmental steps of 5, 20, 35, 65, 80, and 85 for the stress-sharing embryo (Fig. 5B.II), and at steps 1 and 10 for the without-stress sharing embryo. (Fig. 5B.III).

We conclude that stress sharing increases the cognitive light cone of an embryo compared to an embryo without stress sharing (Fig. 5B.I). As development proceeds, the radius of the cognitive light cone decreases, lasting longer for an embryo with stress sharing (Fig. 5B.II), but terminating prematurely in case of an embryo without sharing (Fig. 5B.III).

Sequential morphogenesis causes stress to fluctuate over the course of development.

Next, to observe the spatio-temporal time course of stress during development, we manually broke down the target pattern (smiling face) into a set of sequential targets and observed how stress varied as each target pattern was introduced. Specifically, we broke down the target pattern of the smiling face into a set of discrete components: left eye only > right eye only > eyes only > eyes and smile > complete face. We set each of these components as the target pattern starting from the “left-eye only” target and moving towards the “complete face” target. We introduced a new target only when development had optimized the source pattern to the current target pattern. We identified this point by observing the stress value: as long as it decreased, optimization was treated as in-progress.

**Fig. 6:**
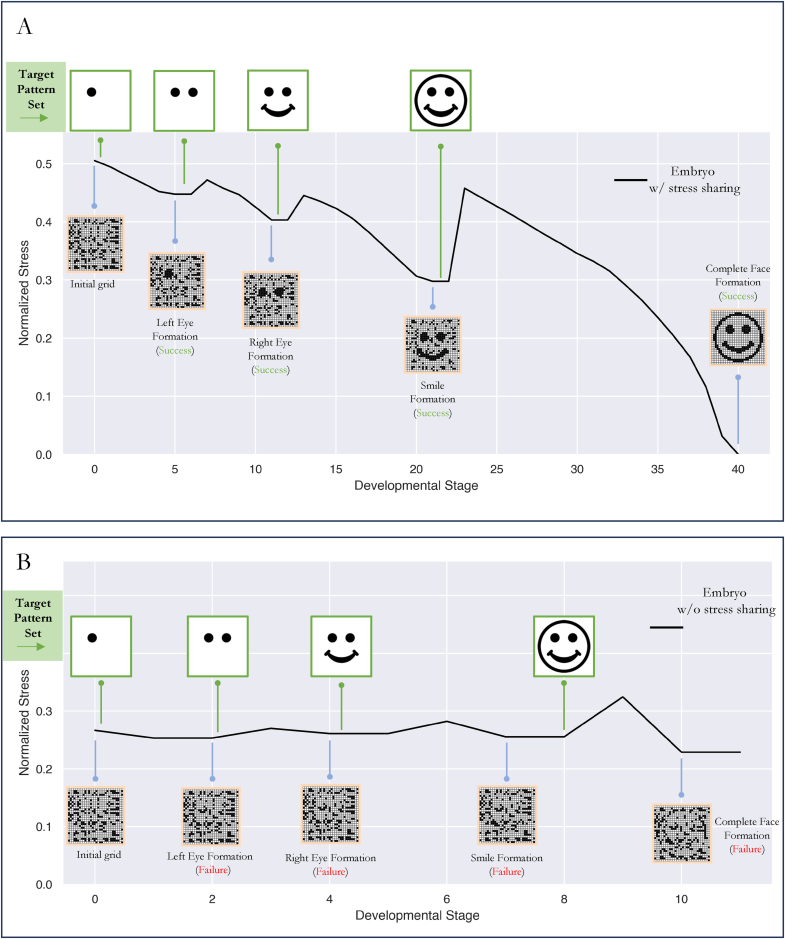
Stress fluctuates for a population w/sharing, oscillating up and down over the course of development, eventually dropping to zero. (A): Normalized stress (over all cells in an embryo) over the course of developmental for a competent population with stress sharing. Initially stress is high owing to the random structure of the embryo's cellular structure. At the same time, a target (left eye pattern) was set as the target to the developmental process. Once the left eye pattern was achieved at generation 5, a new target (one with both, left and right eye) was introduced. Once such a pattern was achieved, a new target (a smile, along with the eyes) was set, and finally, once this pattern was achieved, the entire face was set as target. Throughout such part-by-part morphogenesis, stress continued to drop and rise corresponding to settling at a target pattern, versus moving towards a new pattern. (B): Normalized stress (over all cells in an embryo) for a population without Stress sharing. Stress continues to oscillate as patterns are incrementally introduced at various local minima, but optimization towards these patterns fail because cells are restricted in their movement due to the absence of stress sharing. Stress eventually settles at a non-zero value giving rise to a scrambled pattern. In the absence of stress sharing, such competency relies heavily on mutations to nudge patterns towards a target. Since no mutations were given here, competency did not help.

We took a single embryo of each kind (stress-sharing & without stress-sharing), scrambled their elements randomly from the final target pattern (complete face), and then let them develop towards each sequential partial target pattern, and plotted the average stress within the embryo throughout development (Fig. 6).

We observed that in both kind of grids, stress fluctuated. On introducing a target pattern, stress momentarily increased and then continued to decrease until the current target pattern was attained to the best possible extent by the competency process of the grid. In a stress-sharing embryo, stress progressively decreased until the relevant target pattern was perfectly achieved (Fig. 6A), whereas in embryos without stress sharing, the target pattern was only partially formed due to the constrained cell-movement within its elements (Fig. 6B). Consequently, a stress-sharing embryo was capable of perfectly forming each sequential target pattern and stress was able to reach zero at step 40 (Fig. 6A). The without-sharing embryos however, was unable to form any of the sequential target patterns well. Tiny improvements of ∼1 % in fitness were noticed between each sequential pattern, but progress was limited to movement of less than 55 cells on average (compared to 4500 cells on average for the stress-sharing embryo) and development lasted for 10 steps only (Fig. 6B).

We conclude that sequentially introducing new features increases the average stress, but given enough competency and a communication mechanism (sharing), individual features can be formed perfectly with the corresponding feature's average stress reducing to zero. However, without a communication mechanism, no matter the competency value, cells are restricted in their movement and tend to get stuck in localized patches. Thus, a communication mechanism tied to their homeostatic loop (such as stress-sharing) is key if competency is to have an impact on reorganization capacity.

### 3.5 Stress is not predictive of the target during development

**Fig. 7:**
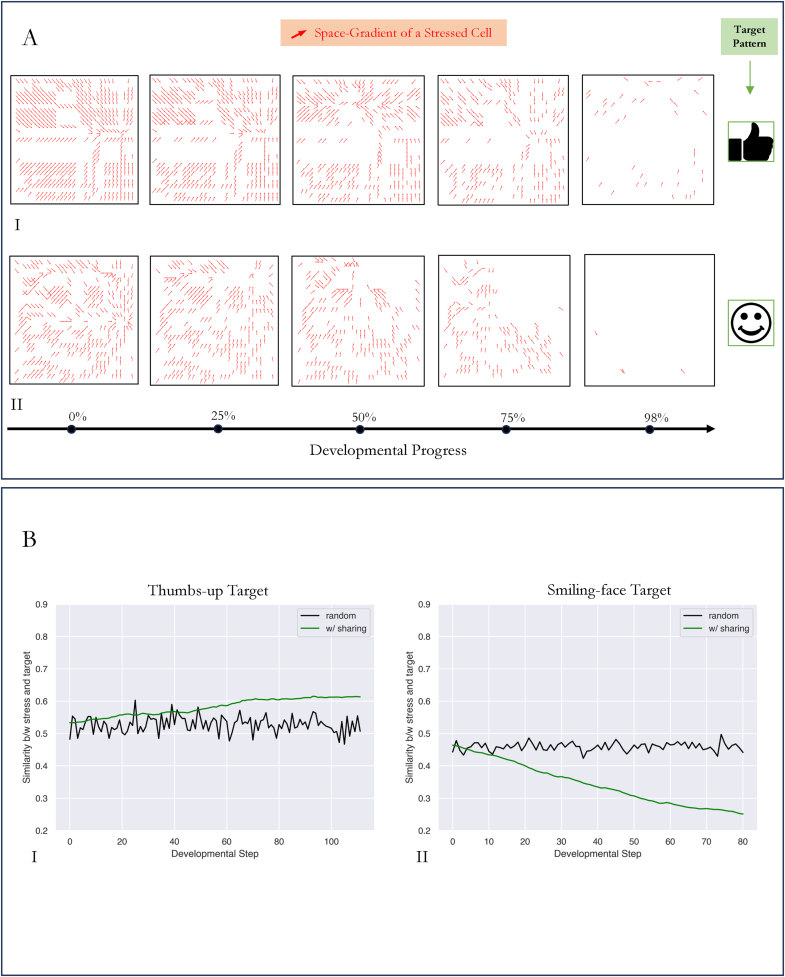
Stress is not predictive of the target pattern being developed. (A): Stress map of an embryo with competent stress sharing ability over several stages of development. Development towards two independent target patterns, a thumb-up and a smiling face, is shown in sequence A.I and sequence A.II respectively. Stress maps shown here are at developmental stages of 0 %, 25 %, 50 %, 75 %, and 98 % to completion. The stress map at any stage is an indication of the directional derivative of stressed-cells with respect to developmental time. Arrows indicate the gradient vector of a corresponding stressed-cell at any stage during development. As development proceeds, stressed cells grow fewer in number, and is reflected by the reduced number of gradient vectors. Note that at 100 % completion, the stress map is an empty field. (B.I): Similarity between the stress map of an embryo w/sharing and the thumbs-up target pattern over the course of development. Similarity increases from 0.53 to 0.61 over 100 developmental stages. In contrast, a randomized stress pattern (which was set up as a control) shows no such trend (black trace), helping differentiate competent sharing based cellular reorganization and random cell movements. (B.II): Similarity between the stress map of an embryo w/sharing and the smiling-face target pattern during development. Similarity decreases from 0.47 to 0.25 over 80 stages of development. A randomized stress pattern was set up as control at each developmental-stage and helped in discerning the effect of competency from the effects of random cell movements (black trace).

When studying minimal models of agency, it's important to ask what can be detected by external observers of the agent vs. what is known only to the system itself (a primitive version of the privacy property of minds). Specifically, since stress is a direct measure of the distance to a morphogenetic setpoint, it might be expected that by observing the stress maps, it would be possible to guess what pattern the cells were attempting to implement – to read the goal state of the system. Could an external observer predict the target pattern being developed by observing fluctuations in stress? We addressed this question by reducing an embryo's reorganization dynamics during development into a series of stress maps. The stress map of an embryo at any time step was a gradient of stress in its two-dimensional grid space. It was obtained by first identifying stressed cells (including those with shared stress) and drawing its space-gradient. By observing the dynamics of such a stress map over the course of developmental time, we sought to determine whether it would reveal the target being formed, as is attempted in the efforts of neural decoding [[60], [61], [62], [63]] (Fig. 7).

We qualitatively assessed predictability of the target by observing the in-time variation of the stress map (Fig. 7A) and observing how visual cues in the stress map allowed predictability, and quantitatively by plotting a similarity metric (see Supplement S3 for details) between the stress map and the target pattern (Fig. 7B) to see if a trend emerges over the course of development.

In this experiment, we employed two different stress-sharing embryos, each of which developed towards two different target patterns (a thumbs up pattern, and a smiling face pattern). We observed that in both cases, during the initial stages of development, the stress map provided partial cues as to the target being formed, but these visual cues disappeared later (at 50 % to completion in both the thumbs-up pattern and the smiling face pattern), providing no definite hint as to the target pattern being formed. (Fig. 7.I and Fig. 7.II). Note that at 100 % completion the stress map would be a blank grid with no stressed cells and therefore no gradients (see Suppl emental Videos 5 and 6 for continuous time evolution of their respective stress maps). Further, it could be that we are observing these “visual cues” precisely because we are primed to see it. To provide a more quantitative assessment we resorted to a similarity metric between the stress map and the target. The similarity metric was chosen to be the inverse l2-distance between the stress map and the target pattern, existing within [0.0, 1.0] with 0.0 indicating no similarity and 1.0 indicating an exact match.

Observing the similarity plot provided a more confusing picture. While for the thumbs-up pattern, the stress maps’ similarity with the target grew from ∼0.53 to ∼0.63, for the smiling face pattern it decreased from ∼0.48 to ∼0.25. Additionally, in each of these plots, we compared the similarity of a random stress map to the target (black trace in Fig. 7B) as control. While the random stress map had a similarity hovering around 0.5, the similarity for the stress sharing embryo varied based on the target being formed.

We conclude that in the absence of prior knowledge about the target pattern being formed, it is unlikely that an external observer can predict the structure being developed by visually inspecting the stress pattern alone. While the similarity metric can indicate the presence of a non-random target being formed, no other predictive information is apparent from either the stress map or its similarity trend with the target during development. Thus, while local stress directly reflects the distance of a cell from its optimal position, the ability to share stress prevents a straightforward inference of the system goal from data available to external observers.

## 4 Discussion

The ability to make desired changes at the system level (repair of complex birth defects, induction of regeneration of appendages, self-assembly of novel biobots, etc.) is hampered by the difficulty of the inverse problem. In most cases, it is not possible to infer changes in the genes determining protein sequence must be made in order to reach a complex anatomical outcome. However, the increasing realization that biological tissues offer high-level interfaces for control, in the same way that learning and communication offers behavioral control without micromanaging individual neurons, suggests novel strategies for their manipulation which are already beginning to be applied in biomedicine [24,25].

The flexible, adaptive aspect of morphogenesis has been modeled as a set of navigation policies within anatomical morphospace [14]. By recasting changes in growth and form as the behavior of cell groups in transcriptional, physiological, and anatomical problems spaces, all of the tools of behavioral science and autonomous system engineering (control theory, cybernetics) can be brought to bear [11,12,64]. Specifically, we have argued that advances in bioengineering and regenerative medicine will require a better understanding the policies with respect to how cellular collectives navigate the landscapes of these problem spaces under different perturbation [11,12,65]. However, the development of such strategies relies on a better understanding of how groups of cells cooperate toward specific outcomes.

A key aspect is that in biology, problem-solving capacity of systems often derives from the competencies of their parts. Stress in this case is a motivating driver of homeostatic adjustments for the cells. It should be noted that some excellent work has been done in the area of understanding how networks (such as gene-regulatory networks) can be guided by stress dynamics to find their way in high-dimensional spaces [[66], [67], [68], [69], [70], [71], [72]].

We constructed a model in which anatomical homeostasis, driven by a set of competent sub-agents, can be examined with respect to a possible role for stress sharing. This model of cellular morphogenesis allowed us to test the hypothesis that sharing of stress, as an indicator of error (distance from the target morphology), would facilitate morphogenesis, be favored by evolutionary processes, and enlarge the effective cognitive light cone of individual cells (increase the scale of goal-directed activity, as befits a mechanism for facilitating collective intelligence dynamics).

### 4.1 Functional implications of stress sharing

We observed that allowing cells to leak stress to their neighbors is beneficial for morphogenesis, allowing the cell collective to reach their target morphology faster and reliably when compared to controls (Fig. 3). This effect persisted across a range of grid sizes, and was due to cells being able to move over longer distances. This movement builds in more freedom and redundancy, which is especially helpful during later stages of morphogenesis when fine-grained improvements are required to finish the pattern (Fig. 5). In effect, the propagation of stress implements a kind of implicit altruism: without having to provide a mechanism for cells to coordinate or care directly about their neighbors’ state, a simple leak mechanism ensures that the stress in one region motivates other cells to work to relieve it. By envisioning leaked stress molecules as features of the microenvironment around stressed cells, our results are also consistent with prior proposals that stigmergy (ability of subunits, such as ants or cells, to leave each other messages in an external medium of the environment) is a universal coordination mechanism [73,74]. We think that this is precisely the kind of simple mechanism biological evolution would seize upon, to increase the collectivity of subunits in a body.

One prediction made by our approach is that cells should leak molecules used as stress markers; indeed, apparently this has been observed for one such molecule – HSP90, a heat-shock protein which also occurs extracellularly [75]. We are currently testing these predictions at the bench, establishing reporter assays for stress and studying how it may spread and with what consequences.

We have previously studied a different but related effect, in which competition for informational and metabolic resources by cells within a single body actually contributes to morphogenetic coordination, such as for example ensuring identical growth of left and right limbs. When cells do not compete (acquiring resources from an infinite reservoir), no cell knows what any other cell did or did not take from that reservoir. However, finite reservoirs allow cells to coordinate their activity because the current state of the reservoir provides information about how much other cells have taken from it. In effect, limiting pools of resources become a kind of global, stigmergic scratchpad, bearing the evidence of activity of cells across distance and enabling them to communicate through this kind of global variable [76,77]. These kind of mechanisms, along with others based on memory propagation between cells [55], contribute to the “cognitive glue” that binds individual cells to common purpose.

### 4.2 Stress and the cognitive light cone

One way to think about unconventional agents such as groups of cells is to model the size of their cognitive light cone: the spatio-temporal distance that represents the size of the largest goal states which their homeostatic mechanisms can pursue. Multicellular development scales these cognitive light cones from the single cell-scale metabolic and transcriptional goals of individual cells to the grandiose, large-scale setpoints of anatomical structures in morphospace. Disorders of this process, such as cancer [78,79], result from a pathological restriction of this radius of concern, when cells treat the rest of the body as external environment. Put another way, cognitive light cone model [55] formalizes the notion of functional care or concern as being defined by the distance at which things that happen can stress an agent to the point of initiating actions to effect change. To explore the hypothesis that stress sharing potentiates collectivity and helps scale morphogenetic goals, we quantified the cognitive light cone of cells with and without stress sharing.

In our analysis, we saw that enabling the sharing of stress initially increases the cognitive light cone of cells, as measured by the farthest distance a given cell can use as the setpoint for its subsequent activity. This is consistent with a role for stress sharing in enlarging “pro-social” behaviors in the tissue. However, during its lifetime, each virtual embryo transitions from large-scale rearrangements to small-scale fine-tuning (similarly to how real development progresses from the major refactoring of gastrulation to local refinement in late stages). While cells *can* take on long-range goals when sharing stress, their *effective* radius of concern shrinks over time, as it becomes less relevant to take distant events into account (Fig. 5B).

These dynamics make an interesting prediction for the biology of aging [80]. While embryonic development involves progressive reduction of error from a prepattern that keeps changing to pull the embryo from stage to stage, eventually the prepattern stops changing and major rearrangements cease – maturity. However, a mature organism is not a static structure because cells inevitably senesce, die, and must be replaced. An adult organism is in equilibrium but its new cells must continuously be placed correctly, not only in anatomical space but also in physiological and metabolic spaces, when they appear. Thus, the homeostatic process continues, but with a very small cognitive light cone that involves only the local microenvironment for resisting tissue degradation. Our model suggests that modulators of stress response may be candidate targets to modulate tissue replacement and aging in vivo.

### 4.3 Stress profiles are private: the 1st person view of an embryo

Our model studied stress propagation, and was constructed and analyzed from a purely functional metric of performance under various conditions as gauged by an external observer (error function). What about the valence of stress – what is it like to be an agent, made of silicon or proteins, at high or low error levels [[81], [82], [83], [84], [85]]? While we don't know what it’s like to be a cell or tissue under stress [36,86,87], stress as a readout of distance from homeostatic equilibrium is a familiar feature of large-scale biology and of our human experience. There have been studies of what it’s like to *be* simulated beings in simplified digital world [57], although we are not aware of studies that focus on the inner worlds of goal-directed artificial agents with stress dynamics.

Here we avoid discussion of the deeper questions of stress as felt prediction error [[88], [89], [90], [91]], metacognitive loops that allow agents to know what they are doing, and associated issues of first-person perspective that is so important to adaptive agents. However, our results do offer one bit of insight into the distinction between the information available to a system vs. what is perceivable by external observers of the system. We found that knowledge of the embryonic stress patterns do not enable one to deduce the morphogenetic goal state it is trying to reach. Such an agent's goals are largely inscrutable to externa observers (such as scientists, conspecifics, parasites, predators, etc.) The agent itself can perceive its homeostatic goal state, because it effectively reaches that target morphology precisely due to the stress distribution. This provides an interesting, yet primitive, example of “privacy” – a feature of brains that hampers efforts at neural decoding [[60], [61], [62], [63]], in which some features of 1st person cognition are inaccessible to 3rd person observers [92,93]. Apparently, even simple systems such as our virtual embryogeny [94,95], with stress sharing, exhibit emergent features of privacy in the context of homeostatic collectives.

### 4.4 Limitations of the model

Our model has a number of limitations that warrant possible extensions in future work. First, it is 2-dimensional – it is not known how such dynamics would play out in three, or higher-dimensional morphospaces. Moreover, our simulation tries to model the dynamics of each and every cell (albeit in a simpler discrete manner) rendering it too expensive to scale. Evolutionary simulations are especially intractable beyond an embryo size of 50x50 on a 256-core machine. A reductionist approach to stress sharing might reduce the simulation burden and enable further scaling. Regardless, massively parallel computing architectures will need to be considered to study a bio-realistic numbers of cells.

Our simulation takes place in a fixed, discrete grid, which cannot itself deform as cells move. As such, it does not capture some of the recurrent complexity of morphogenesis in which the action landscape of cells is modified by other cells’ actions. And, we only simulated two different cell states, reducing the possible complexity. Furthermore, we did not include multi-scale stress – future models will study the interplay of stress at different levels of organization in the same virtual embryo, from the molecular to the cell-, tissue-, and whole organism level, as new dynamics and problem-solving behaviors may emerge when homeostats are coupled [13,96].

## 5 Conclusion

Homeostasis, and mechanisms which expand the size of the goal states towards which it works, is a central concept for understanding agency [[97], [98], [99]], regenerative competency [11,12], and evolution [[50], [54],[100], [101], [102]]. Thus, future empirical work must identify mechanisms by which stress is sensed and distributed within and across groups of cells. Existing tools of molecular genetics and biophysics can then be deployed for loss- and gain-of-function studies of the role of stress in morphogenetic competency, to test the predictions of our model in vivo. More broadly, it may be that stress and geometric frustration [103] are a truly interdisciplinary concepts, reaching far beyond the conventional notions of psychological stress and highlighting a central symmetry across fields ranging from the computational mathematics and physics of Ising models and Hopfield networks [104,105] to the remarkable capabilities of regulative morphogenesis [5,54] and even the epigenetics of transgenerational inheritance [[106], [107], [108]]. Stress is a good candidate for a central invariant across active agents of highly diverse scale and composition. Feedback between computational models and wetlab biology, focused around the primary concept of the causal geometry of stress, offer the exciting potential to unify and advance aspects of the field of diverse intelligence in ways that benefit fundamental questions of cognitive science, robotics engineering, and biomedical disorders of homeostatic cellular collectives.

## CRediT authorship contribution statement

**Lakshwin Shreesha:** Writing – review & editing, Writing – original draft, Software, Methodology, Investigation, Formal analysis, Conceptualization. **Michael Levin:** Writing – review & editing, Writing – original draft, Supervision, Project administration, Methodology, Funding acquisition, Formal analysis, Conceptualization.

## Declaration of competing interest

The authors declare the following financial interests/personal relationships which may be considered as potential competing interests:Michael Levin reports financial support was provided by 10.13039/501100011730Templeton World Charity Foundation Inc. for support of co-author Lakshwin Shreesha. If there are other authors, they declare that they have no known competing financial interests or personal relationships that could have appeared to influence the work reported in this paper.
