# Supplementary material for: Stress sharing as cognitive glue for collective intelligences: A computational model of stress as a coordinator for morphogenesis
Source: Biochem Biophys Res Commun. 2024 Oct 30;731:None. doi: 10.1016/j.bbrc.2024.150396 (PMC11356093; doi:10.1016/j.bbrc.2024.150396)
Supplement: Multimedia component 1 [file mmc1.docx]

Supplementary information for

“Stress Sharing as Cognitive Glue for Collective Intelligences: a computational model of stress as a coordinator for morphogenesis”

**Supplement 1**

The general theme of our paper concerns the in-silico evolution of two-dimensional artificial embryos capable of undergoing morphogenetic development during their lifetime. In this section we provide a brief description of our experimental setup and provide rationale for having designed it as such.

Design Choices

Artificial embryo in our framework was a two-dimensional square matrix. A matrix data structure was chosen because it provided us with an opportunity to draw parallels with real embryos (albeit in two-dimensions): cells of the matrix could be treated analogous to biological cells. It also helped simplify simulations by restricting cell dynamics into a set of discrete actions (int0 one of 8 possible different cardinal directions).

The matrix data structure was also ideal to represent patterns of different kinds. For instance, a matrix with a scrambled set of cell types could be designed with a cellular re-rearrangement subroutine which could re-arrange its cells into a distinctive pre-set pattern. Thus, the matrix’s’ simple structure as well as its ability to help us study morphogenetic pattern formation made it the ideal substrate for our simulations.

In this paper, our main objective was two-fold: 1. to observe how different kinds of developmental processes impacted evolutionary dynamics and, 2. To probe why one developmental process was better than another.

It may help to define what we mean by development here, because it differs from biology. Here, development as a process of cellular re-organization only. We focus only on the re-organization process, choosing to ignore cell-growth and other chemical processes which make real development complex.

We resorted to initializing embryos with two cell types (0 and 1) because it was sufficient to form a morphological pattern in two-dimensions. Ignoring growth meant that each embryo had to have the same number of cell types as that of the target. To achieve this, we scrambled the target pattern using a random seed across both axes and treated the resulting scrambled matrix as an embryo which had just been initialized. This helped us avoid trying to design a developmental process which had to balance the number of cell-types, and we could just focus on reorganization.

Once initialized, each embryo was passed through our genetic algorithm: which was an iterative sequential process of the following steps: 1. Development, 2. Selection, and 3. Mutation.

A description of each of these processes is provided in the main text. We made a number of choices about which aspects of the model to focus on in terms of biological realism. Given the discrete nature of our 2-D grid, capturing the idea that “cells can slide or squeeze around each other” was difficult. Thus, rather than implementing realistic deformable cell biomechanics in 3D space, we simply abstracted cell movement as a kind of “tunnel”
that enables a third pseudo dimension through which cells can move.

In the following section, we provide pseudo-code for each of the stages which occur during a single stage of evolution. We begin with the genetic-algorithm. The structure it provides helps put the rest of the sub-routines in context.

**Supplement 2: Algorithms**

**Algorithm 1** Genetic Algorithm

Initialize a population of $M$ embryos, each of size $N$, with development marker $G$

Initialize empty lists $g\_fitness$ and $p\_fitness$

**for** generation = $1, N\_gen$ **do**

**for** genotype = 1, M **do**

store **fitness** (genotype, target) in a temporary list, $temp\_g$

phenotype = $development$ (genotype, competency_value)

store $fitness$ (phenotype, target) in a temporary list, $temp\_p$

**end for**

// store embryo with the best fitness

store $max$(temp_p) in $p\_fitness$

store the $argmax$(temp_p)^th^ entry of $temp\_g$ in $g\_fitness$

selected_genotypes = $selection$ (genotypes, temp_p)

genotypes = $mutation$ (selected_genotypes)

**end for**

plot ($g\_fitness$, $p\_fitness$)

**Algorithm 2** Initialization

Get target matrix, $T$; population size, $M$; and population type, $G$

//get embryo size

N = $size$ (T)

//set empty population tensor

P = $empty$ (M, N, N)

//set population by scrambling $T$, using $M$ different seeds.

rng = $random\_generator$ (seed)

**for** i = 1, M **do**

embryo = scramble(T, axis = (0, 1), rng)

Add $embryo$ to population tensor, $P$

**end for**

Return $P$

**Algorithm 3** Development (single embryo)

Get embryo, $E$; its development marker, $G$, target, T; and competency value, $cv$

set $competency\_used$ and $distance\_travelled$ to 0.0

**while** $competency\_used$ < $cv$ **do**

1. identify stressed positions, $stress\_pos$ by comparing $E$ with the target, $T$

2. identify unstressed positions, $fixed\_pos$ by comparing $E$ with the target, $T$

3. for each $fixed\_pos$ in $E$, get its corresponding $neighbors$ (in all 8 directions)

from the target, $T$;

4. store each $neighbor’$s [cell-value, $(x,y)$ coordinate] as *neighborhood information.*

5. for each $fixed\_pos$ in $E$, ignore recorded *neighborhood information*

if $neighbor$ is unstressed.

6. for each recorded $neighbor$ of $fixed\_pos$, send a radially diminishing

distress signal (with initial strength 1.0) to all $stress\_pos$’s in $E$with a

cell-value as that noted in its *neighborhood information.*

7. record distress signals received from multiple $fixed\_pos$ in each $stress\_pos$

8. pick a random $stress\_pos$

9. consult its corresponding distress signal and recover target ($x,y$ location)

10. swap from $stress\_pos$ to the chosen $(x,y)$ coordinate in the direction of

shortest path

11. if G == “sharing”:

swap normally

update $cv$ by counting number of swaps

12. if G == “not sharing”:

block if a swap is encountered with a $fixed\_pos$

update $cv$ by counting number of swaps

continue

//An analogy of the above steps : stressed positions are analogous to

//wounded positions, and fixed positions to surrounding normal tissue.

//The normal surrounding tissue requests assistance by calling cells of the correct

//type to move to the injured site and relieve its stress.

return E

**Algorithm 4** Selection

Get genotypes of a population, $P$ and their respective phenotypic fitness post development, $p\_fitness\_list$; both with a size of $M$

1. $sorted\_indices$ = argsort ($p\_fitness\_list$, order = descending)

2. $selected\_indices$ = pick the first 10% from the $sorted\_indices$ list

3. $selected\_genotypes$ = pick those genotypes from P whose indices match those

in $selected indices$

return $selected\_genotypes$

**Algorithm 5** Mutation

Get selected population, $p\_selected$, with a size $10\% * M$;

**while** (size ($p\_selected$) < M) **do**

**for** embryo in $p\_selected$ **do**

with probability $pr\_mutation$ do

new_embryo = swap (embryo, random_first_index, random_second_index)

add $new\_embryo$ to $p\_selected$

check size of $p\_selected$; break if $> M$

return $p\_selected$

**Supplement 3: Experimental details**

Experiment 1: Evolution of a single population with different developmental markers G

Developmental marker (G) types: with-sharing, without-sharing, hardwired

Target pattern: smiling face

Population size (M) = 100

Embryo size (N) = 30

N_generations = 1000

Competency value (cv) = $(N^{2} *0.75) * 7$

N_runs = 10

Mutation rate (pr_mutation) = 0.3

Experiment 2 : Evolution of populations with three different embryo sizes.

Developmental marker (G) types: with-sharing, without-sharing, hardwired

Target pattern: smiling face

Population size (M) = 100

Embryo size (N) = 20 or 30 or 50

N_generations = 1000

Competency value (cv) = $(N^{2} *0.75) * 7$

N_runs = 10

Mutation rate (pr_mutation) = 0.3

Experiment 3, 4: Development of a single embryo

Developmental marker (G) types: with-sharing, without-sharing

Target pattern: smiling face

Population size (M) = 1

Embryo size (N) = 30

Competency value (cv) = $inf$

Repeatedly pass the single embryo through development until fitness is maximum.

Experiment 5: Development of a single embryo towards two different targets

Developmental marker (G) types: with-sharing, without-sharing

Target pattern: smiling face or thumbs-up emoji

Population size (M) = 1

Embryo size (N) = 30

Competency value (cv) = $inf$

Repeatedly pass the single embryo through development until fitness is maximum.

Similarity Metric for Experiment 5

- Similarity between the stress map and the target was computed throughout the course of development to check whether a correlation existed between the two.
- The presence of such correlation would have allowed a third-person observer to predict the target pattern being formed by looking only at the stress map changing over development-time.
- Similarity was quantified as the inverse l2-distance between the stress map and the target (1.0 – l2 (stress-map, target)); with a value of 0.0 indicating no similarity and 1.0 indicating maximum similarity.
- Note: the stress map was a binary 2D matrix where the stressed cells (i.e., those which were out of position with respect to the target) were indicated with a 1.0 and unstressed cells (those which were in their correct positions with respect to the target) were indicated with a value of 0.0.

**Plots**

- In each of our evolutionary experiments we tracked the embryo with the highest phenotypic fitness and plot its phenotypic fitness, genotypic fitness, competency value, and the average distance travelled by its cells during a generation. These plots are reproduced in figures 3A, 4, and 5A.
- To observe how stress evolved over developmental time we plot the space-gradient of stress during the course of development of a single embryo (Figure 7). The space-gradient was a two-dimensional map with each point in the map corresponding to a single cell in the embryo. Those cells which were stressed were depicted by vectors represented as red arrows. Each vector’s magnitude was normalized to maintain legibility; however, its direction conveyed positional information about the movement of the stressed cell. Specifically, it indicated the direction towards which the stressed cell intended to move towards during the developmental stage.
- We provide code to reproduce all our figures at:

<https://github.com/lakshw1n/CellularCompetency2D/tree/master/visualisation>

- Video files of the space-gradient map, and target pattern formation are attached as additional supplementary files along with this document.

**Supplement 4**

Hardware

Evolutionary experiments were run on the tufts university HPC cluster on ten compute nodes, each with the following specifications:

cpu = intel ® Xeon® Gold @ 3.10Ghz

cores count = 100

Memory/cpu = 250mb

We ran each evolutionary experiment on a single node. There were three developmental markers to choose from (sharing, without-sharing, and hardwired), and also three different grid sizes (20, 30, and 50), so a total of nine different experiments could be run at any single time. We used the additional node to run non-evolutionary (development exclusive) experiments.

In each node, we parallelized the developmental stage of each embryo as an independent task on each core. We had the option of choosing between parallelizing over number of runs or the developmental process and we choose parallelizing over developmental runs because we noticed a speedup in it.

On average, an evolutionary experiment with embryos of size 30, and a developmental marker, G of “with-sharing” took about 40 hours to complete 1000 iterations of our genetic algorithm; whereas with a developmental marker, G of “without-sharing” took about 70 hours to complete the same number of iterations.

Increasing the embryo size to 50, pushed the “with-sharing” marker to take around 100 hours, and the without sharing marked population to take around 150 hours. We concluded that beyond an embryo size of 55, it would be impossible to run 1000 iterations of our GA in realistic time.

Software

Our code is open source and can be found here: <https://github.com/lakshw1n/CellularCompetency2D>

All our code was written in python. When we initially began, we did not expect scaling from one embryo size to another to be an issue. Indeed, we designed the stress-based system not accounting for the role of scale, and also without foreseeing how expensive our design choices would be when we scaled embryo sizes beyond 50.

In our simulations, we modelled the dynamics of each and every cell in an embryo and looking back this was a poor decision because it created a maximum embryo size beyond which we could not scale. This was observed to be around 55 from our experiments.

A different design is required if we are to scale stress-based development to larger grid sizes or even to three-dimensions. To this end, a reductionist approach might be the ideal way forward.
